# Double-Wound O-Ring Retraction for Chylothorax Surgery in Dogs

**DOI:** 10.3390/ani13162567

**Published:** 2023-08-09

**Authors:** Piotr Trębacz, Jan Frymus, Marek Galanty

**Affiliations:** Department of Small Animal Diseases with Clinic, Institute of Veterinary Medicine, Warsaw University of Life Sciences—SGGW, Nowoursynowska 159c, 02-776 Warsaw, Poland; marek_galanty@sggw.edu.pl

**Keywords:** elastic wound retractor, chylothorax, dog

## Abstract

**Simple Summary:**

Chylothorax is a life-threatening disease resulting in accumulation of chyle in the thoracic cavity due to thoracic duct leakage. In many cases, the only treatment is surgical tying of the damaged duct with a thread or by placing a ligation clip. However, such an operation is technically demanding as it requires suitable exposure of the lymphatic system structures. Stay sutures and/or classical (metal) tissue retractors, as used so far, provide sufficient exposure of the thoracic duct only after several manipulations during the surgery, which prolongs the operation and increases infection risk. In this communication, we present our impressions of using o-ring elastic wound retractors (O-WRs) instead of classical retraction in three large breed dogs. We conclude that the O-WRs provide a static circumferential vision of the operation field and good access to the thoracic duct. The usage of other surgical instruments is not hampered by the O-WRs. In turn, the elastic rings are not damaged by the metal instruments. In addition to classical retraction, the O-WRs, once placed in the diaphragmatic area, do not require further manual retraction.

**Abstract:**

A suitable wound retraction is crucial for open surgical treatment of chylothorax in dogs. A single paracostal approach for transabdominal/transdiaphragmatic thoracic duct ligation and cisterna chyli ablation is an effective procedure. For the procedure, the use of stay sutures and handheld or automatic soft tissue retractors is recommended. However, it is often necessary to adjust the retractors several times during the surgery to provide sufficient exposure of the thoracic duct. This prolongs the operation and increases infection risk. In this report, we describe the modified application of two o-ring elastic wound retractors (O-WRs) in three large breed dogs with idiopathic chylothorax. We conclude that the O-WRs provide a static circumferential vision of the operation field and good access to the cisterna chyli, especially to the thoracic duct. The usage of other surgical instruments is not hampered by the O-WRs, and the elastic rings are not damaged by the metal instruments. Once placed in the diaphragmatic area, indeed, they do not require further manual retraction.

## 1. Introduction

Chylothorax is a life-threatening disease resulting in the accumulation of chyle in the pleural cavity. In small animals, chylothorax has several causes, including trauma, lung lobe torsion, cardiac disease, diaphragmatic hernia, and neoplasia. In many cases, the cause cannot be determined and the condition is termed as idiopathic chylothorax (IC). Medical and surgical treatment of chylothorax has been reported with varying degrees of success in dogs. The basic surgical procedure is thoracic duct ligation (TDL), which prevents the flow of chyle to the pleural cavity. TDL can be performed in the traditional way by laparotomy and transdiaphragmatic thoracotomy, thoracotomy, or a minimally invasive procedure (thoracoscopic techniques). Additional surgical procedures, including cisterna chyli ablation (CCA) and subphrenic pericardiectomy (SP), have been incorporated with TDL in various combinations to improve the postoperative success rate. A combination of TDL and CCA has a high success rate for the resolution of IC [1,2]. In the current study, Mayhew et al. found that in dogs without evidence of constrictive pericardial physiology (CPP), TDL alone was associated with a very good prognosis for the treatment of IC. In the absence of CPP, the additional benefit of pericardiectomy in the treatment of IC is questionable. Since the results do not differ between the different techniques, and since pericardiectomy carries a risk of ventricular fibrillation, a TDL and a CCA is crucial [3].

Each of the abovementioned procedures (except thoracoscopic techniques) have been described with separate approaches: a right ninth/tenth intercostal thoracotomy for TDL and median celiotomy for CCA [1,2]. Performing multiple surgical approaches increases the risk of postoperative complications. Additionally, any required repositioning and repreparation prolongs the anesthesia. In 2011, Staiger and colleagues [2] proposed a single paracostal approach for both TDL and CCA. In order to improve the approach to the thoracic duct and the cisterna chyli, the authors recommended using stay sutures and tissue retractors (handheld and automatic).

An o-ring elastic wound retractor (O-WR) can be an alternative to stay sutures and metal retractors. It is a single-use, cylinder-shaped device made of two semi-rigid rings attached by a thin flexible polymer membrane. This device provides atraumatic wound retraction, maximizes the exposure, minimizes the incision size, and thus reduces the risk of wound infection [4,5]. To obtain a quick and easy retraction, the flexible internal green ring is introduced into the wound and the stiffer external white ring is rolled to tighten the membrane until the two rings are adjacent to each other.

**Figure 1:**
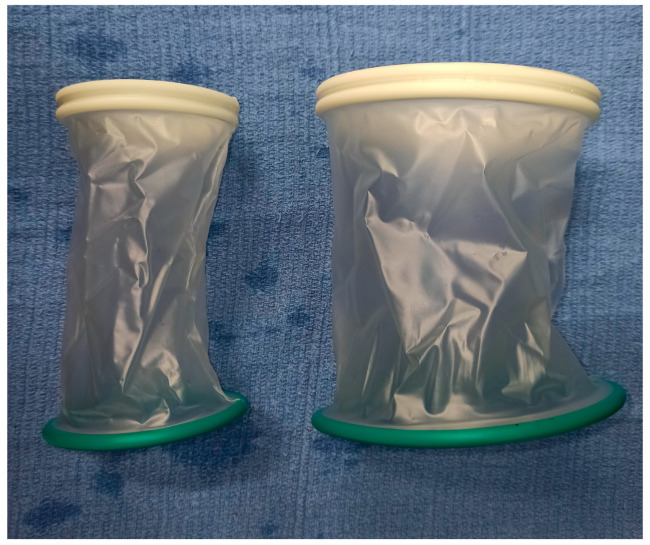
The 80 × 90 × 150 mm (**left**) and 120 × 130 × 150 mm (**right**) o-ring elastic wound retractors are comprised of two semi-rigid polymer rings attached by a flexible polymer membrane.

Currently, in cases of IC in dogs, the single paracostal approach is usually performed in our clinic. In this communication, we present our impressions after applying O-WRs (Figure 1) during this procedure in order to improve the surgical approach.

## 2. Materials and Methods

Three client-owned large breed dogs were referred to the Small Animal Clinic of Warsaw University of Life Sciences with a suspicion of IC.

### 2.1. Diagnosis of IC

The clinical symptoms identified in the preoperative evaluation were tachypnea and anorexia. In all patients, IC was confirmed by physical examination, complete blood count, serum biochemical analysis, echocardiography examination, pleural effusion analysis following thoracocentesis, and computed tomography with lymphography.

Informed consent was obtained for client-owned animals to be included in this study.

### 2.2. Surgery

Patients were premedicated intramuscularly with a mixture of dexmedetomidine (Dexdomitor, Orion Pharma, Wels, Austria) at a dose of 0.005 mg/kg and methadone (Comfortan, Dechra, Bladel, The Netherlands) at a dose of 0.2 mg/kg. General anesthesia was induced with propofol (initial dose 1 mg/kg to effect; Proposure, Axience, Pantin, France) and maintained with isoflurane (Isovet, Piramal Healthcare, Surrey, UK) in oxygen. Intraoperative analgesia was provided by fentanyl boluses (0.0025 mg/kg). Postoperatively to reduce pain, the dogs received buprenorphine (Bupaq Multidose, Orion Pharma, Wels, Austria) at a dose of 0.02 mg/kg every 8 h for the next 3 days, and meloxicam (Metacam, Boehringer Ingelheim, Germany) at a dose 0.1 mg/kg for the next 5 days.

**Figure 2:**
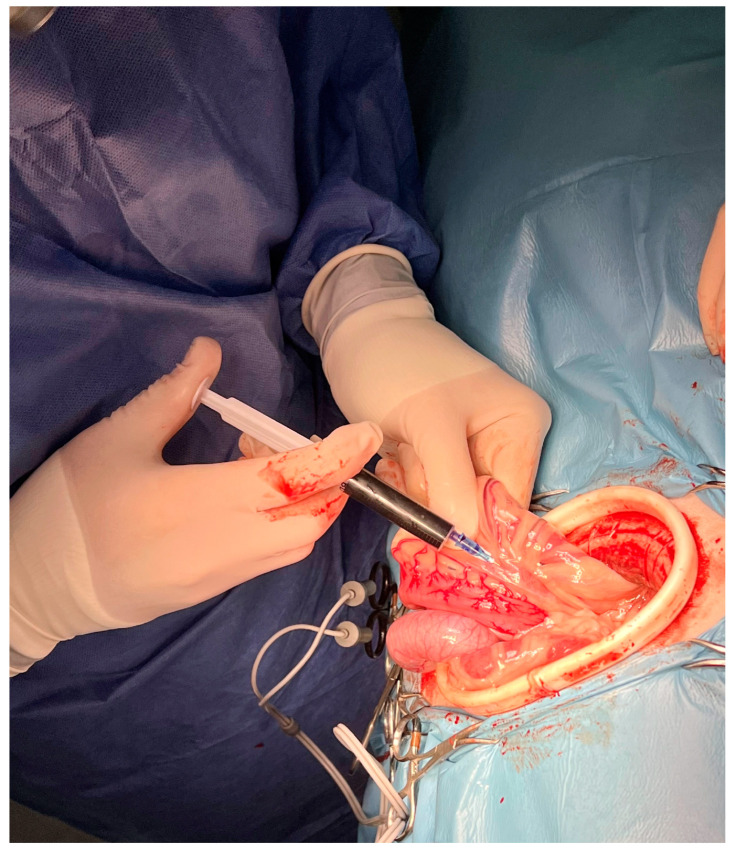
Right-sided paracostal laparotomy after wound retraction by an 120 × 13 × 150 elastic retractor. After manual extraction of a small intestine loop, fractioned injections of methylene blue were performed into mesenteric lymph node. Male Labrador retriever (dog no. 1), head on the bottom.

**Figure 3:**
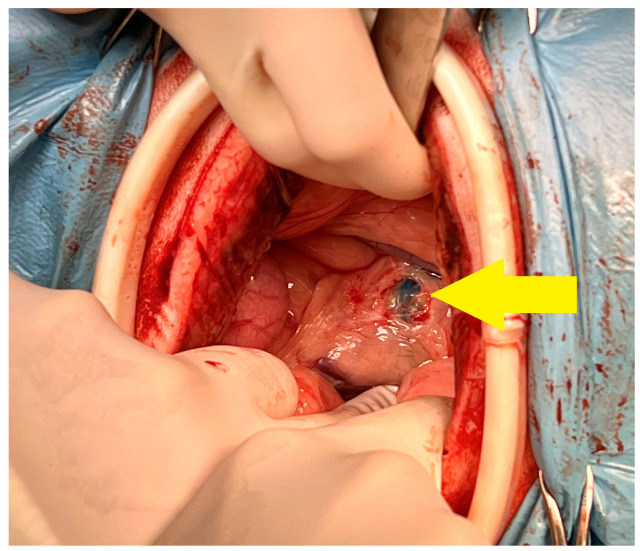
Preparation of cisterna chyli (Yellow arrow) filled by methylene blue. Dog no. 1.

Each dog was positioned in left lateral recumbency. A right paracostal abdominal incision was performed. The abdominal wound was retracted by an elastic 120 × 130 × 150 mm O-WR (Ring Protect^TM^, Grena, Brentford, UK), intended for wounds 5–10 cm long. Next, a loop of small intestine was manually extracted, and fractioned injections of methylene blue were given into a mesenteric lymph node (Figure 2) in order to visualize the lymphatic vessels. After identification of the cisterna chyli by dissection around the abdominal aorta (Figure 3) and bipolar cauterization and separation of the right phrenicoabdominal artery and vein, a right circumcostal transdiaphragmatic thoracotomy was performed. Before isolation of the thoracic duct, the sympathetic trunk and major splanchnic nerve were identified and preserved.

**Figure 4:**
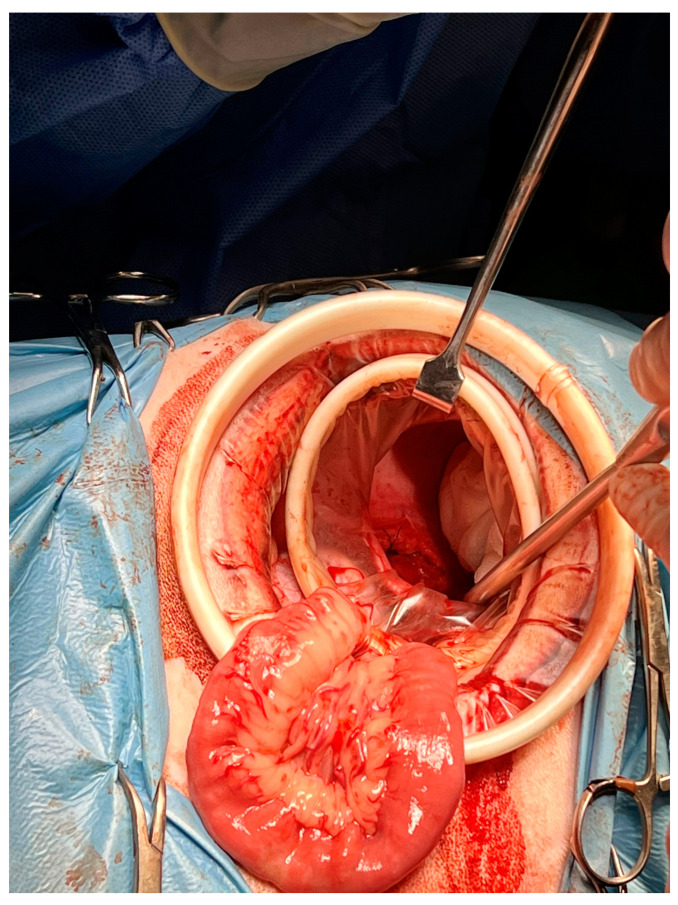
The second 80 × 90 × 150 mm elastic retractor is inserted transdiaphragmatically into the thoracic cavity. This provided a good operative view of the right pleural space. Dog no. 1.

**Figure 5:**
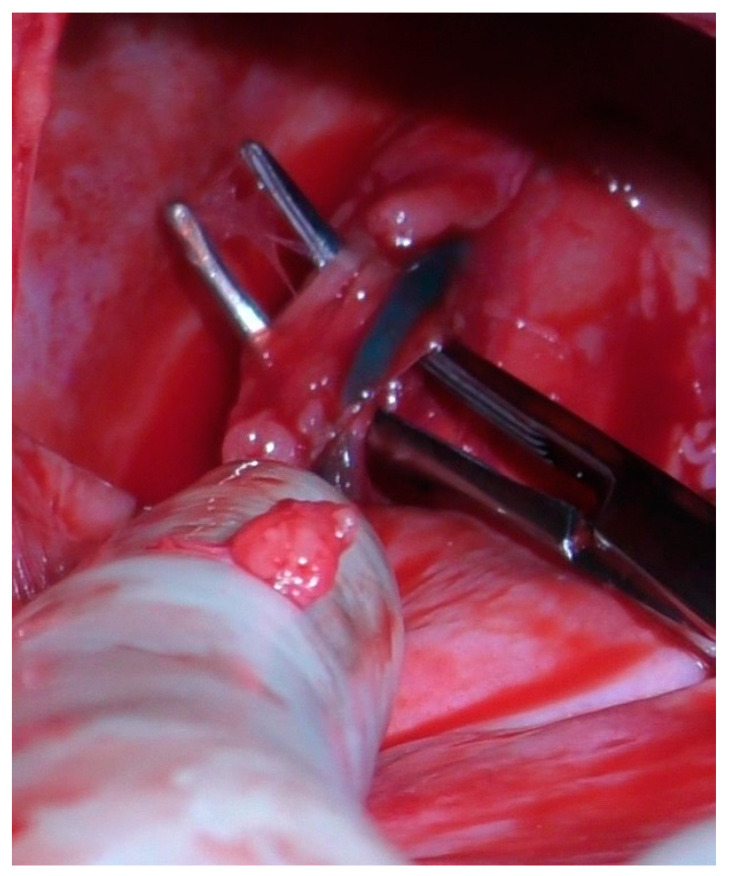
Preparation of the thoracic duct stained by methylene blue. Dog no. 1.

The diaphragmatic incision was retracted by an elastic 80 × 90 × 150 mm O-WR (Ring Protect^TM^, Grena, Brentford, UK) intended for wounds 2.5–6 cm long (Figure 4). After isolation of the thoracic duct (Figure 5), two ligations of the duct with 3-0 poliamid suture material (Amifil M, Sinpo, Poznań, Poland) were performed. Then, the wall of the cisterna chyli was excised and omentum was placed within its lumen. After the introduction of a chest tube and removal of wound retractors, the diaphragm and abdominal wall were closed in a typical manner. Postoperatively, the dogs were administered supportive and analgesic therapy.

## 3. Results

The signalment and outcomes of the three patients with IC are summarized in Table 1. The average age was 5.3 years (range 4–7). There were two males and one female. The average body weight was 31.6 kg (range 29–34). Chest tubes were removed after 2 days once pleural effusion had decreased to 2 mL/kg/day. In the early postoperative period, a self-limiting abdominal chylous effusion was noted in dogs 1 and 3. In dog 3, abdominocentesis was performed two times between days 2 and 4 postoperatively. Chylous pleural effusion resolved in all dogs within the median time of 4 months (range 2–6).

In all dogs, the O-WRs were easy to insert. The circumferential traction provided by the retractors enabled very good views of the operative field. In all dogs, depending on the need, during the surgery it was possible to manipulate the diaphragmatic O-WR and move the surgical window by inserting a handheld retractor under the outer ring of the device. This ensured that there was no direct tension on the diaphragm, thereby protecting the wound.

## 4. Discussion

Since TDL alone has an unsatisfactory cure rate, various methods such as CCA, SP, omentalization of the pleural cavity, and shunt tube placement have been combined with TDL [2,6]. However, the optimal combination of surgical techniques is still being debated. The CCA is a promising surgical procedure for combination with TDL. A prospective comparative study of CCA combined with TDL showed that this procedure resulted in an 83% cure rate [2,7]. Although recent studies have been published about the use of O-WRs in many surgical procedures in humans and animals (maxillo-facial surgery, intraoral robotic surgery, thyroid surgery, cesarean section, minithoracotomy, intestinal surgery, etc.) [4,8,9], the results described in our report provide evidence that a double-wound retraction by two O-WRs can be successfully performed as an adjunct procedure during surgical treatment of canine chylothorax. The combined transabdominal and transdiaphragmatic approach is technically demanding and requires suitable exposure of intraabdominal and intrathoracic structures of the lymphatic system. The use of stay sutures and tissue retractors (handheld and automatic), as proposed by Staiger and colleagues [2], is difficult and in our opinion seldom provides sufficient exposure of the thoracic duct via lateral transdiaphragmatic approach. The tension on traction sutures and positioning of the retractors must be often manipulated during the surgery to reach adequate exposure, which prolongs the operation, increasing infection risk. The advantages of the technique described in this report include the easy insertion of the retractors into the abdominal and thoracic cavity. Furthermore, once in place, no further adjustment of the retractors is required. Robb and colleagues [10] shared their similar experience with a minimally invasive approach to the aorta in human patients. Having the O-WR in position, static circumferential vision was achieved, providing good access to the cisterna chyli and especially the thoracic duct in all treated dogs. The placement of two elastic retractors did not prevent the use of other surgical instruments in the operating field. We did not notice any damage of the elastic retractors by the usage of metal instruments. The O-WR sheath does not tear easily because it is made of polyurethane, which is very resistant, despite its thickness of only 0.08 mm. Igai et al. [11] reported that the retractor sheath was not torn by contact with the fragments of broken ribs, metal implants, and instruments.

Compared to previous surgeries using traction sutures, hooks, and automatic retractors, we did not notice a significant change in the length of the surgical wounds and the duration of the time of the surgical procedures. An important change was the significant improvement in access to the thoracic duct during the transdiaphragmatic thoracotomy.

The O-WRs are available in different sizes according to the length of the surgical incision. We have successfully performed the same procedure on smaller dogs and even cats in the past. In our study with large breed dogs, relatively small elastic retractors for incisions between 2.5–6 cm and 5–10 cm length were suitable to provide an excellent view of the surgical field.

## 5. Conclusions

The O-WRs provide a static circumferential vision of the operation field, and good access to the cisterna chyli and especially to the thoracic duct. The usage of other surgical instruments is not hampered by the O-WRs, and neither the elastic rings are damaged by the metal instruments. The O-WRs, once placed in the diaphragmatic area, do not require further manual retraction.

## Figures and Tables

**Table 1:** Clinical data and outcome of the surgical treatment of idiopathic chylothorax in 3 dogs.

Dog No.	Breed	Age (Years)	Gender	BW (kg)	Clinical Signs	Time Post TDL-CCA (Months) *	Outcome
1	Labrador retriever	7	male	32	Tachypnea, anorexia	2	Good, postoperative self-limiting chyloabdomen
2	German Shepherd	5	male	34	Tachypnea, anorexia	4	Excellent
3	Golden retriever	4	female	29	Tachypnea, anorexia	6	Good, postoperative self-limiting chyloabdomen

* at the time of writing.

## Data Availability

Not applicable.
